# Association Between Bioimpedance-Determined Metabolic Age and MASLD Risk Scores in Spanish Workers

**DOI:** 10.3390/metabo15050343

**Published:** 2025-05-21

**Authors:** Ignacio Ramírez-Gallegos, Carla Busquets-Cortes, Hernán Paublini, Ángel Arturo López-González, Emilio Martínez-Almoyna-Rifá, Pedro Juan Tárraga López, José Ignacio Ramírez-Manent

**Affiliations:** 1 ADEMA-Health Group, University Institute of Health Sciences Research (IUNICS), 07009 Palma, Balearic Islands, Spain; i.ramirez@eua.edu.es (I.R.-G.); c.busquets@eua.edu.es (C.B.-C.); h.paublini@eua.edu.es (H.P.); emilio@mompra.com (E.M.-A.-R.); pjtarraga@sescam.jccm.es (P.J.T.L.); jignacioramirez@telefonica.net (J.I.R.-M.); 2 Faculty of Dentistry, University School ADEMA, 07009 Palma, Balearic Islands, Spain; 3 Balearic Islands Institute of Health Research (IDISBA), Balearic Islands Health Research Institute Foundation, 07010 Palma, Balearic Islands, Spain; 4 Balearic Islands Health Service, 07010 Palma, Balearic Islands, Spain; 5 Faculty of Medicine, University of Castilla la Mancha, 02008 Albacete, Castilla-La Mancha, Spain; 6 Faculty of Medicine, University of the Balearic Islands, 07010 Palma, Balearic Islands, Spain

**Keywords:** non-alcoholic fatty liver disease, metabolic age, bioimpedance, MASLD risk scores, lifestyle factors, occupational health, metabolic health assessment

## Abstract

**Background**: Metabolic dysfunction-associated steatotic liver disease (MASLD) is a prevalent liver disorder with significant metabolic implications. Metabolic age, determined through bioimpedance analysis, has emerged as a potential indicator of overall metabolic health. The objective of this study is to evaluate the association between metabolic age and MASLD risk scores in a cohort of Spanish workers. **Methods**: A cross-sectional study was conducted on 8590 Spanish workers who underwent annual occupational health examinations between 2019 and 2020. Metabolic age was determined using bioelectrical impedance analysis, and the Avoidable Lost Life Years (ALLY) index was calculated as the difference between their metabolic and chronological age. MASLD risk was assessed using various validated scales, including the Fatty Liver Index (FLI), Hepatic Steatosis Index (HSI), Zhejiang University Index (ZJU), Fatty Liver Disease Index (FLD), and Lipid Accumulation Product (LAP). A multinomial logistic regression analysis was performed to examine the association between metabolic age and MASLD risk scores, adjusting for sociodemographic and lifestyle variables. **Results**: Higher metabolic age values were observed in individuals with greater MASLD risk across all evaluated scales. The mean metabolic age was consistently lower in women compared to men, and these differences were statistically significant (*p* < 0.01). Multinomial logistic regression analysis revealed that the strongest associations with increased metabolic age were found for MASLD risk scores, physical inactivity, and poor adherence to the Mediterranean diet. ROC curve analysis demonstrated a high predictive capacity for the FLD (AUC: 0.935 in women and 0.917 in men) and FLI (AUC: 0.900 in women and 0.833 in men), with high Youden index values. **Conclusions**: Metabolic age is significantly associated with MASLD risk, suggesting its potential as a non-invasive biomarker for identifying individuals with a higher risk for metabolic liver disease. Lifestyle factors, including physical activity and dietary patterns, play a crucial role in modulating metabolic age, highlighting the importance of targeted interventions for MASLD prevention. Further research is warranted to validate metabolic age as a prognostic tool in MASLD risk assessment.

## 1. Introduction

Metabolic dysfunction-associated steatotic liver disease (MASLD), formerly known as non-alcoholic fatty liver disease (NAFLD), is a widespread chronic liver condition affecting over 30% of the global population, with a substantial impact on public health [1] and a significant economic burden on healthcare systems [2]. It is defined as the excessive accumulation of fat in the liver (hepatic steatosis) in the absence of significant alcohol consumption and other secondary causes of liver disease [3]. MASLD arises from complex interactions involving cardiometabolic, environmental, and genetic factors, with insulin resistance and adipose tissue dysfunction playing central roles. The condition encompasses a spectrum of liver pathologies ranging from simple steatosis to its most severe form, previously termed non-alcoholic steatohepatitis (NASH) [4], but now referred to as metabolic dysfunction-associated steatohepatitis (MASH), as well as hepatic fibrosis [5], and in some cases, cirrhosis [6] and hepatocellular carcinoma [7]. Due to its strong association with metabolic syndrome [8] and other non-communicable chronic diseases, investigating MASLD is essential for developing effective preventive and therapeutic strategies. Despite growing research efforts and a deeper understanding of its pathophysiology, disease progression and treatment responses remain heterogeneous. This led to a global initiative—the Nomenclature Development Initiative—to adopt a more accurate and less stigmatizing terminology. The goal was to reflect the underlying causes of the disease more clearly, improve diagnostic clarity, raise awareness, and promote better resource allocation for research and healthcare [9].

The prevalence of MASLD has increased exponentially in recent decades, paralleling the rise in obesity and type 2 diabetes. Recent studies estimate that between 25% and 30% of the global population is affected by MASLD [10], with higher rates in Western countries and certain regions of Asia [11]. Factors such as population aging [12], physical inactivity [13], and diets rich in refined sugars and saturated fats [14] have been identified as key contributors to this emerging epidemic. Additionally, differences in MASLD prevalence have been observed among ethnic groups and sexes, with a higher predisposition in individuals of Hispanic ancestry and a lower prevalence among individuals of African descent [15], suggesting the involvement of genetic factors in its pathogenesis.

The development of MASLD has been described under the “two-hit” hypothesis. In the first hit, insulin resistance promotes an increase in hepatic lipogenesis and a reduction in fatty acid oxidation, leading to triglyceride accumulation in hepatocytes [16]. In the second hit, various factors, such as oxidative stress [17], chronic inflammation [18], and mitochondrial dysfunction [19], contribute to disease progression to more severe stages, such as MASH and fibrosis. Moreover, the role of the gut microbiome has gained relevance in MASLD pathophysiology, as alterations in gut microbiota can promote an inflammatory state and increased intestinal permeability, exacerbating liver damage [20].

The clinical implications of MASLD extend beyond the liver, as it has been identified as an independent risk factor for cardiovascular disease [21], type 2 diabetes [22], and chronic kidney disease [23]. In fact, cardiovascular disease is the leading cause of morbidity and mortality in MASLD patients, highlighting the need for a comprehensive risk assessment in these individuals [24]. At the hepatic level, progression to advanced fibrosis and cirrhosis can lead to liver failure and the need for transplantation [25]. Additionally, MASLD has been associated with an increased risk of developing hepatocellular carcinoma, even in the absence of cirrhosis, emphasizing the importance of monitoring and early detection in high-risk populations [26].

Since liver biopsy remains the gold standard for diagnosing MASH and fibrosis [27], various risk scores have been developed to stratify patients without requiring invasive procedures. These include the Fibrosis-4 index (FIB-4) [28], MASLD Fibrosis Score (NFS) [29], Body Mass Index-Age-Insulin Resistance Score (BAAT) [30], Fatty Liver Index (FLI) [31], Hepatic Steatosis Index (HSI) [32], Zhejiang University Index (ZJU) [33], Fatty Liver Disease Index (FLD) [34], and Lipid Accumulation Product (LAP) [35]. These scoring systems have demonstrated a strong predictive capacity for identifying patients at higher risk of disease progression, facilitating clinical decision making and the selection of candidates for interventional studies.

The concept of metabolic age has emerged as an alternative biomarker for assessing an individual’s overall health status in comparison to their chronological age. It is calculated based on various physiological parameters, including body mass index, body composition determined by bioelectrical impedance, and other metabolic factors [36]. A metabolic age higher than one’s chronological age indicates a deteriorated health status and an increased risk of metabolic diseases, including obesity, type 2 diabetes, and MASLD.

The interest in metabolic age lies in its ability to provide a comprehensive evaluation of biological aging and the impact of lifestyle factors on health. Recent studies have shown that a higher metabolic age is correlated with increased systemic inflammation [37] and mitochondrial dysfunction [38], both of which are key processes in MASLD pathophysiology. Furthermore, metabolic age has been proposed as a useful indicator for identifying individuals at risk of developing chronic liver diseases, potentially enabling early intervention and personalized prevention strategies [39].

Metabolic age (MA) reflects the functional status of an individual’s metabolism and has emerged as a key indicator of overall health. It provides a more accurate assessment than chronological age, enabling the early detection of metabolic disturbances even in individuals who appear clinically healthy. Its utility is particularly relevant in the prevention of chronic diseases such as type 2 diabetes, hypertension, dyslipidemia, metabolic dysfunction-associated steatotic liver disease (MASLD), and cardiovascular disease, facilitating personalized interventions to improve metabolic health and prevent comorbidities.

MA is a concept used to evaluate the efficiency with which the body performs its basic metabolic functions by comparing an individual’s physiological status with population averages across different chronological age groups [40]. Its estimation is based on a range of physiological parameters, including body composition [41], basal metabolic rate (BMR) [42], body fat percentage, and other indicators of functional status [43].

BMR refers to the minimum energy required by the body at rest to maintain vital functions. It declines by approximately 1–2% per decade in adulthood, primarily due to the progressive loss of muscle mass, which is gradually replaced by adipose tissue. Although BMR can be estimated using formulas that incorporate age, sex, weight, and height, these methods fail to distinguish between fat mass and lean mass, limiting their accuracy in determining MA.

Bioelectrical impedance analysis (BIA) is a non-invasive technique that improves BMR estimation by differentiating between fat mass, fat-free mass, skeletal muscle mass, and total body water [44,45,46,47]. Unlike conventional methods based on weight or body mass index (BMI), BIA provides a more precise assessment of body composition, which is essential for accurate MA calculation. Skeletal muscle, being metabolically active, has a significantly greater influence on BMR than fat mass, which has only a minimal effect (2–3%) [48]. Studies conducted by TANITA have shown that BMR estimated through BIA is more accurate than that derived from weight or BMI alone [49]. Furthermore, BIA has proven particularly useful in patients with MASLD, allowing for the early identification of metabolic imbalances and supporting personalized interventions [50]. BIA has outperformed traditional anthropometric methods and shows good agreement with dual-energy X-ray absorptiometry (DXA) for estimating body fat percentage [51,52,53]. More recently, Cretescu et al. (2025) confirmed a strong correlation between BIA-derived values and actual body fat percentage, further supporting its clinical utility in metabolic health assessment [54].

MASLD represents a growing global health challenge with implications extending beyond the liver to multiple organ systems. Understanding its epidemiology, pathophysiology, and clinical consequences is crucial for developing effective management strategies. In this context, the use of risk scores and biomarkers such as metabolic age could improve disease detection and prevention, enabling more personalized patient care.

The objective of this study is to evaluate the association between different MASLD risk scores and metabolic age values in a large cohort of Spanish workers.

## 2. Material and Methods

### 2.1. Participants

**Figure 1:**
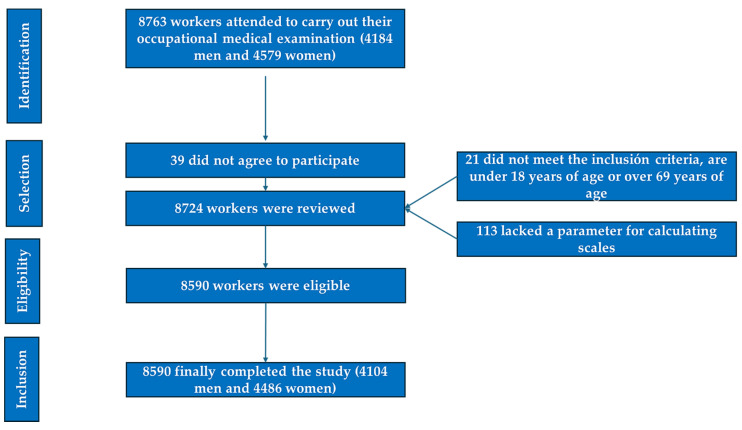
PRISMA diagram illustrating the participant selection process for this study.

A cross-sectional, descriptive study was conducted involving a total of 8590 Spanish workers based in the Balearic Islands. The sample comprised individuals who underwent their annual occupational health examination between January 2019 and December 2020 at a workplace health and risk prevention service. This service provides coverage to various companies operating across multiple sectors, including healthcare, public administration, hospitality, retail, transportation, education, industry, and cleaning services. Further details on participant selection and study flow can be found in Figure 1.

Inclusion criteria:

- Individuals aged between 18 and 69 years;
- Voluntary participation in the study;
- Provision of informed consent for the use of personal data in epidemiological research;
- Active employment with one of the companies included in the study, without being on temporary disability leave at the time of participation;
- Not a habitual alcohol drinker;
- Not suffering from known liver disease, thyroid disease, celiac disease, or drug addiction.

Exclusion criteria:

- Age below 18 or above 69 years.
- Lack of employment within the participating companies;
- Refusal to participate in the study;
- Refusal to grant consent for the use of personal data in epidemiological research;
- Absence of a required parameter necessary for scale calculations;
- Habitual alcohol drinker;
- Known liver disease;
- Hypothyroidism;
- Hypopituitarism;
- Celiac disease;
- Drug addiction;
- Inborn errors of metabolism;
- Patients on antiretroviral therapy.

### 2.2. Variable Determination

The data collection process was carried out by healthcare professionals affiliated with the occupational health services of participating companies. The following methods were employed:

- Medical History Assessment: A comprehensive clinical history was obtained, covering sociodemographic variables such as age, sex, social class, smoking status, physical activity levels, and adherence to the Mediterranean diet;
- Anthropometric and Clinical Measurements: Parameters including height, weight, waist and hip circumference, and both systolic and diastolic blood pressure were recorded;
- Laboratory Analyses: Blood lipid profile and glucose levels were measured.

### 2.3. Anthropometric Measurements

To ensure the reliability and consistency of the data, standardized measurement techniques were applied. Height and weight assessments were performed using an SECA 700 scale and an SECA 220 stadiometer, with participants wearing only light clothing, following international anthropometric evaluation standards established by the International Society for the Advancement of Kinanthropometry (ISAK) [55]. Measurements were documented in centimeters and kilograms.

Waist circumference was measured using an SECA tape, positioned midway between the last rib and the iliac crest, parallel to the floor, with participants standing in a relaxed posture. Hip circumference was measured in a similar manner, at the widest point of the buttocks.

### 2.4. Clinical Measurements

Blood pressure was assessed using an OMRON-M3 digital sphygmomanometer. Participants were instructed to remain seated with their backs supported, resting for at least ten minutes before measurement. The assessment was conducted with the arm positioned at heart level, ensuring no recent intake of food, alcohol, tobacco, or caffeinated beverages in the preceding hour. The cuff was placed 2–3 cm above the elbow crease, ensuring a secure but non-restrictive fit. Three consecutive measurements were taken at one-minute intervals, with the final reading calculated as the mean of the three measurements.

### 2.5. Laboratory Analyses

Blood samples were collected via venipuncture following a 12 h fasting period.

The samples were processed as follows: An 8.5 mL BD SST II Vacutainer serum tube with gel separator (reference BD 366468) was used. The samples were transported to the laboratory in a refrigerated container (at between 5 and 10 degrees Celsius). Upon arrival, the samples were centrifuged within two hours of collection and immediately analyzed using an automated analyzer [56,57]. LDL was calculated using the Friedewald formula, except in cases with triglycerides ≥ 400 mg/dL, for which direct measurement was used [58]. All bio-chemical variables are reported in milligrams per deciliter (mg/dL).

### 2.6. Risk Assessment Scales

- Adherence to the Mediterranean Diet: Assessed using the PREDIMED questionnaire, a validated 14-item instrument in which each question is assigned a score of zero or one. A total score of nine or higher indicates strong adherence to the Mediterranean diet [59].
- Physical Activity Levels: Evaluated using the International Physical Activity Questionnaire (IPAQ), a self-reported survey capturing physical activity over the previous seven days [60].
- Smoking Status: Individuals who had smoked at least one cigarette per day (or its equivalent) in the past 30 days, or who had quit smoking within the last 12 months, were classified as smokers. Non-smokers included individuals who had abstained from smoking for at least one year or had never smoked.
- Socioeconomic Classification: Defined according to the Spanish Society of Epidemiology guidelines based on the 2011 National Classification of Occupations [61].
  ○ Class I: Senior executives, directors, and university-educated professionals.
  ○ Class II: Intermediate professionals and self-employed individuals.
  ○ Class III: Manual laborers.
- Metabolic Age: Determined using a TANITA MC-780 S MA bioimpedance meter (TANITA Corporation, Tokyo, Japan).
  Avoidable Lost Life Years (ALLY): Calculated as the difference between metabolic age and chronological age. Previous studies suggest that a metabolic age at least 12 years lower than chronological age is associated with reduced cardiovascular risk. ALLY classification [62]:
  ○ Low: Difference of less than three years;
  ○ Normal: Difference of three to eleven years;
  ○ High: Difference of 12 years or more;
  ○ A metabolic age exceeding one’s chronological age by 12 years or more was considered a high-risk threshold.

The risk of MASLD was determined by applying different scales:

- Fatty Liver Index (FLI) [63] FLI = (e^0.953 × log (triglycerides) + 0.139 × BMI + 0.718 × log (GGT) + 0.053 × waist circumference − 15.745)^/(1 + e^0.953 × log (triglycerides) + 0.139 × BMI + 0.718 × log (GGT) + 0.053×waist circumference − 15.745)^ × 100. FLI values above 60 are considered high risk;
- Hepatic Steatosis Index (HSI) [64] HSI = 8 × AST/ALT + BMI + 2 if diabetic and + 2 if female. Values above 36 are considered high risk;
- Zhejiang University Index (ZJU index) [65] ZJU = BMI + glycemia (mmol L) + triglycerides (mmol L) + 3 AST/ALT + 2 if female. Values above 38 are considered high risk;
- Fatty Liver Disease Index (FLD) [66] FLD = BMI + triglycerides + 3 × (AST/ALT) + 2 × hyperglycemia (present = 1; absent = 0). Values above 37 are considered high risk;
- Lipid Accumulation Product (LAP) [67] = Men. (waist (cm) − 65) × (triglycerides (mMol)) and Women: (waist (cm) − 58) × (triglycerides (mMol)). Values above 42.7 are considered high risk.

### 2.7. Statistical Analysis

A descriptive analysis was performed on categorical variables, presenting their frequency distributions. For normally distributed quantitative variables, means and standard deviations were calculated. Student’s *t*-test was used for comparing means, while the chi-square test was employed for comparing proportions. The dependent variable, ALLY, was categorized into three groups. Given that it consists of more than two categories, a multivariate analysis stratified by age groups was conducted to evaluate the effect of each studied variable within each stratum. Independent variables were selected based on their statistical and biological relevance as indicated in the literature review. The multivariate analysis was performed using multinomial logistic regression, with odds ratio calculations and the Hosmer–Lemeshow goodness-of-fit test applied for model evaluation. Statistical analyses were conducted using SPSS version 29.0, with a significance threshold of 0.05.

## 3. Results

The anthropometric and clinical characteristics of the study participants are summarized in Table 1. The analysis included a total of 8590 individuals, comprising 4104 men (47.8%) and 4486 women (52.2%). The mean age of the participants was slightly above 41 years, with the majority falling within the 30–49 age range. The analysis of anthropometric, clinical, and biochemical parameters revealed significantly lower values in the female participants across all measured variables. Most participants were classified within social class I. Regarding smoking status, approximately 15% of both the men and women were current smokers. Physical inactivity was observed in 25.9% of the male participants and 35.1% of the female participants. Furthermore, more than half of the participants in both genders adhered to the Mediterranean diet.

Table 2 presents the mean values of ALLY metabolic age according to the risk scale values for MASLD. The mean values of ALLY metabolic age are higher in individuals with a greater risk of MASLD. The mean values are consistently lower in women. In all cases, the differences are statistically significant (*p* < 0.01).

Table 3 presents the results of the multinomial logistic regression. All independent variables (sex, age, social class, tobacco consumption, Mediterranean diet, physical activity, and MASLD risk scales) are associated with the values of ALLY metabolic age. Among these, the variables showing the strongest associations (the highest odds ratios) are the MASLD risk scales, physical activity, and the Mediterranean diet.

**Figure 2:**
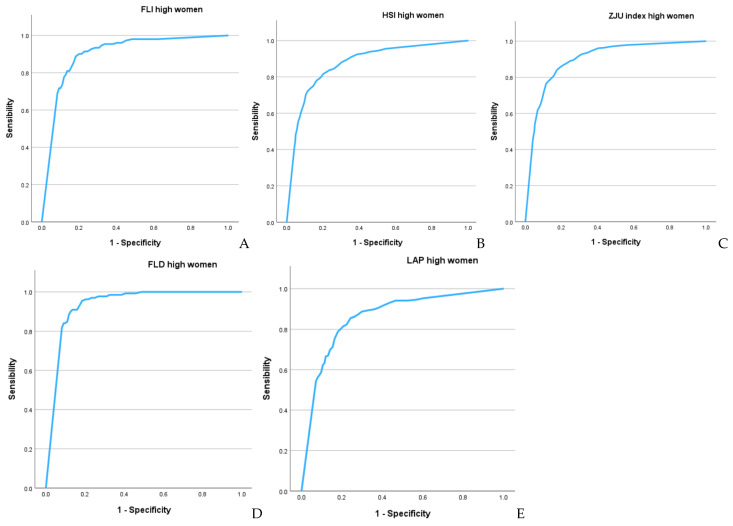
ROC curves in women: (**A**) FLI, (**B**) HSI, (**C**) ZJU, (**D**) FLD, (**E**) LAP.

**Figure 3:**
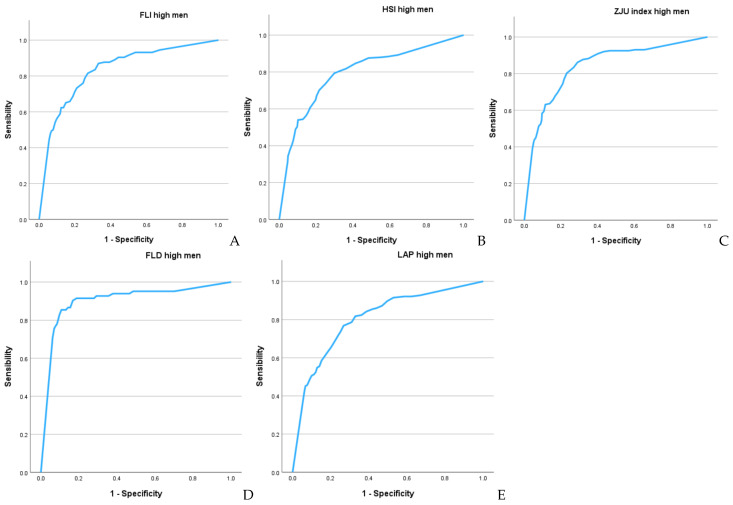
ROC curves in men: (**A**) FLI, (**B**) HSI, (**C**) ZJU, (**D**) FLD, (**E**) LAP.

Figure 2 and Figure 3, along with Table 4, present the results of the ROC curves. The areas under the curve for both sexes show very high values (which are higher in women), with the highest values observed for FLD (0.935 in women and 0.917 in men) and the FLI (0.900 in women and 0.833 in men). The Youden index values are also high, particularly for FLD (0.759 in women and 0.736 in men) and the FLI (0.669 in women and 0.602 in men).

## 4. Discussion

Considering the results of this study, we can affirm that there is an association between metabolic age values and the values of different MASLD risk scales.

Metabolic dysfunction-associated steatotic liver disease (MASLD) is a highly prevalent liver pathology worldwide, characterized by the accumulation of fat in the liver in the absence of excessive alcohol consumption. Its development and progression are strongly influenced by metabolic factors, including obesity [68], insulin resistance [69], and dyslipidemia [70]. Recently, the concept of metabolic age has emerged as a comprehensive indicator of an individual’s metabolic status, potentially providing insights beyond traditional risk markers. Metabolic age is a composite measure that integrates various physiological signs, including body composition, fat distribution, and basal metabolic rate (BMR), into a single, easily interpretable index that reflects an individual’s overall metabolic health status. This integrative nature allows for a more comprehensive assessment of underlying metabolic disturbances that may not be apparent through conventional indicators. Therefore, metabolic age has been proposed as a non-invasive biomarker with particular utility for cardiometabolic risk stratification in both clinical and population-based settings. In this context, the objective of this discussion is to analyze the relationship between metabolic age and MASLD, as well as to explore the influence of sociodemographic factors and health habits on metabolic age.

In this research, we have confirmed that individuals with higher values across all analyzed MASLD risk scales exhibited higher metabolic age values. These findings are consistent with various studies demonstrating that an elevated metabolic age compared to chronological age is associated with an unfavorable metabolic profile, including a high body mass index (BMI) [71], insulin resistance [42], and increased visceral adiposity [72]. These metabolic conditions also represent key factors in the pathogenesis of MASLD. It has been identified that individuals with a metabolic age exceeding their chronological age present a higher risk of developing hepatic steatosis, suggesting that metabolic age could serve as a prognostic marker for disease progression [73].

One of the fundamental mechanisms linking metabolic age to MASLD is mitochondrial dysfunction [74] and increased oxidative stress [75]. It has been postulated that a higher metabolic age is accompanied by a reduction in oxidative phosphorylation efficiency, contributing to the accumulation of reactive oxygen species (ROS) in hepatocytes, thereby promoting inflammation and hepatic fibrosis [76]. Additionally, insulin resistance, a common feature among individuals with a higher metabolic age, plays a critical role in hepatic lipid accumulation through the activation of de novo lipogenesis.

Recent studies have suggested that metabolic age assessment could be used for risk stratification in MASLD patients. A recent investigation demonstrated that an elevated metabolic age was significantly associated with higher levels of alanine aminotransferase (ALT) and aspartate aminotransferase (AST), hepatic biomarkers used to assess disease severity [77]. Furthermore, the relationship between increased metabolic age and hepatic fibrosis suggests a direct impact on the progression of MASLD to more advanced stages, such as metabolic dysfunction-associated steatohepatitis (MASH) and cirrhosis [78].

Chronological age is a natural determinant of metabolic age; however, the disparity between the two can be influenced by various sociodemographic factors, as observed in this study. In particular, sex has proven to be a relevant variable in modulating metabolic age, both in this research and in others [36]. Hormonal differences between men and women can influence body composition [79], fat distribution [80], and insulin sensitivity [81], which in turn may impact metabolic age. It has been observed that men tend to have a higher metabolic age compared to women, possibly due to lower estrogenic protection against insulin resistance and oxidative stress [36].

Socioeconomic status has also been identified as a key determinant of metabolic age in this study. It is well known that individuals of lower socioeconomic status often have limited access to resources that promote a healthy lifestyle, such as a balanced diet and opportunities for regular physical activity [82]. Additionally, chronic stress associated with economic instability may promote metabolic dysfunction through neuroendocrine mechanisms, such as the hyperactivation of the hypothalamic–pituitary–adrenal (HPA) axis [83].

Considering the results of this research, several health habits appear to modulate metabolic age and, consequently, influence the development of MASLD. Tobacco consumption, for instance, is associated with a higher metabolic age due to its negative impact on endothelial function, increased oxidative stress, and promotion of chronic inflammation. These mechanisms also contribute to MASLD progression [84].

Conversely, adherence to a Mediterranean diet has been associated with a lower metabolic age, both in this study and in others [85]. This dietary pattern, characterized by a high intake of monounsaturated fatty acids, polyphenols, and fiber, contributes to improved insulin sensitivity [86] and reduced systemic inflammation [87]. Studies have demonstrated that the Mediterranean diet can decrease hepatic fat accumulation and mitigate MASLD progression [88].

Physical activity is another crucial factor in the modulation of metabolic age, as evidenced by the findings of this study. Regular physical exercise improves cardiorespiratory fitness [89], optimizes mitochondrial function [90], and reduces visceral adiposity [91]. It has been demonstrated that physically active individuals have a significantly lower metabolic age and a reduced risk of developing MASLD [92].

In our study, we assessed MA using BIA, a non-invasive, rapid, and cost-effective technique that enables a more accurate estimation of BMR. This method has proven useful not only for evaluating body composition but also as an indirect indicator of an individual’s overall metabolic status. Our findings revealed a significant association between a higher metabolic age and elevated scores on various MASLD risk scales. Specifically, participants with higher values on the analyzed indices (such as the FLI, HSI, ZJU, FLD, and LAP) also showed higher MA values, suggesting a greater degree of underlying metabolic impairment.

These results are consistent with those reported by Choi et al. (2022) [93], who identified a significant relationship between BIA-derived parameters and the presence of hepatic steatosis. In their study, the authors proposed BIA as a valuable screening tool for fatty liver disease in both men and women. In this context, our results further support the hypothesis that BIA-derived metabolic age is not only effective in characterizing the functional state of metabolism but may also serve as an early marker of hepatic metabolic risk. This has important clinical implications, as it could aid in the early identification of individuals at risk of MASLD and guide personalized interventions aimed at improving metabolic health and preventing progression to more advanced stages of liver damage.

Adherence to a balanced Mediterranean diet, regular engagement in physical activity, weight management, and smoking cessation are modifiable lifestyle behaviors that, according to our findings, significantly influence metabolic age and the risk of developing metabolic dysfunction-associated steatotic liver disease (MASLD). These results support the role of comprehensive lifestyle interventions as effective strategies for improving metabolic health and preventing MASLD. Promoting such habits may offer substantial public health benefits, particularly in populations at increased risk of metabolic and hepatic disorders.

## 5. Conclusions

Metabolic age has emerged as a non-invasive and clinically valuable indicator of overall metabolic health, with particular relevance in the early detection of risk for metabolic dysfunction-associated steatotic liver disease (MASLD). An MA higher than one’s chronological age typically reflects an underlying metabolic impairment, making it a potential screening tool. Sociodemographic factors and lifestyle habits influence MA, highlighting the need for integrated preventive strategies. Assessing MA enables the early identification of individuals at risk, thereby facilitating personalized interventions aimed at improving metabolic health and preventing progression to more advanced stages of liver damage.

## Figures and Tables

**Table 1:** Characteristics of the population.

	Men n = 4104	Women n = 4486	
	Mean (SD)	Mean (SD)	*p*-Value
Age (years)	41.6 (10.6)	41.5 (10.5)	0.492
Height (cm)	175.8 (7.2)	162.5 (6.1)	<0.001
Weight (kg)	81.2 (14.8)	63.9 (13.6)	<0.001
Waist circumference (cm)	89.8 (12.5)	77.0 (12.0)	<0.001
Hip circumference (cm)	101.8 (8.7)	99.6 (10.9)	<0.001
Systolic blood pressure (mmHg)	128.6 (13.3)	117.2 (14.1)	<0.001
Diastolic blood pressure (mmHg)	79.9 (10.2)	74.9 (9.9)	<0.001
Glycemia (mg/dL)	93.4 (17.8)	88.9 (12.6)	<0.001
Total cholesterol (mg/dL)	191.8 (36.0)	189.0 (34.8)	<0.001
HDL-cholesterol (mg/dL)	49.2 (11.3)	59.5 (12.8)	<0.001
LDL-cholesterol (mg/dL)	124.0 (54.6)	113.8 (30.7)	<0.001
Triglycerides (mg/dL)	107.8 (69.4)	81.5 (46.3)	<0.001
GGT (UI)	31.5 (30.0)	18.5 (15.9)	<0.001
AST (UI)	24.4 (17.3)	18.2 (7.7)	<0.001
ALT (UI)	29.3 (34.9)	17.3 (13.4)	<0.001
	%	%	*p*-value
18–29 years	15.5	16.8	0.005
30–39 years	27.8	25.1	
40–49 years	32.7	34.4	
50–59 years	19.0	19.7	
60–69 years	5.0	4.0	
Social class I	57.1	50.8	<0.001
Social class II	20.2	23.8	
Social class III	22.7	25.4	
Non-smokers	84.5	84.2	0.348
Smokers	15.5	15.8	
No physical activity	25.9	35.1	<0.001
Physical activity 1–3 days/week	27.0	26.5	
Physical activity more 3 days/week	47.1	38.4	
Mediterranean diet not followed	44.5	41.6	<0.001
Mediterranean diet followed	55.5	58.4	

GGT Gamma-glutamyl transpeptidase. AST Aspartate transaminase. ALT Alanine transaminase.

**Table 2:** Mean values of metabolic age according to values of sociodemographic variables, healthy habits, and MASLD risk scales by sex.

		Men			Women	
Metabolic Age	n	Mean (SD)	*p*-Value	n	Mean (SD)	*p*-Value
FLI low	2206	−10.3 (6.1)	<0.001	3645	−8.7 (8.5)	<0.001
FLI moderate	971	−3.0 (9.7)		478	6.3 (9.4)	
FLI high	1107	7.1 (9.6)		361	11.7 (6.7)	
HSI normal	2518	−10.2 (6.9)	<0.001	3268	−10.5 (6.6)	<0.001
HSI high	1766	3.6 (10.6)		1216	6.5 (9.6)	
ZJU normal	3102	−9.5 (7.5)	<0.001	3443	−10.2 (7.0)	<0.001
ZJU high	1182	6.1 (9.5)		1041	8.1 (8.5)	
FLD normal	3700	−7.2 (9.0)	<0.001	4175	−7.3 (9.6)	<0.001
FLD high	584	12.2 (6.8)		309	13.0 (5.2)	
LAP normal	3276	−7.1 (9.4)	<0.001	3994	−7.4 (9.7)	<0.001
LAP high	1008	5.2 (10.4)		490	8.5 (9.0)	

FLI Fatty Liver Index. HSI Hepatic Steatosis Index. ZJU Zhejian University. FLD fatty liver disease. LAP Lipid Accumulation Product.

**Table 3:** Multinomial logistic regression.

	MA High	MA High	MA High	MA High	MA High
	OR (95% CI)	OR (95% CI)	OR (95% CI)	OR (95% CI)	OR (95% CI)
Women	1	1	1	1	1
Men	1.27 (1.20–1.34)	1.18 (1.14–1.23)	1.15 (1.10–1.21)	1.10 (1.07–1.14)	1.23 (1.18–1.29)
18–29 years	1	1	1	1	1
30–39 years	1.13 (1.10–1.17)	1.20 (1.16–1.25)	1.15 (1.11–1.20)	1.11 (1.08–1.15)	1.16 (1.10–1.23)
40–49 years	1.29 (1.24–1.35)	1.38 (1.31–1.45)	1.28 (1.21–1.35)	1.22 (1.16–1.28)	1.30 (1.20–1.41)
50–59 years	1.48 (1.38–1.57)	1.56 (1.50–1.63)	1.46 (1.38–1.54)	1.40 (1.31–1.50)	1.48 (1.35–1.61)
60–69 years	1.79 (1.64–1.94)	1.75 (1.66–1.85)	1.69 (1.58–1.80)	1.66 (1.51–1.81)	1.63 (1.49–1.78)
Social class I	1	1	1	1	1
Social class II	1.79 (1.46–2.12)	1.67 (1.50–1.84)	1.84 (1.57–2.11)	1.49 (1.35–1.632)	1.63 (1.48–1.79)
Social class III	2.33 (1.95–2.71)	1.89 (1.70–2.09)	2.22 (1.82–2.62)	1.99 (1.64–2.35)	2.43 (2.10–2.77)
Non-smokers	1	1	1	1	1
Smokers	1.12 (1.10–1.15)	1.24 (1.18–1.30)	1.29 (1.20–1.39)	1.09 (1.06–1.11)	1.17 (1.10–1.24)
Physical activity more 3 days/week	1	1	1	1	1
Physical activity 1–3 days/week	1.96 (1.64–2.28)	1.88 (1.56–2.20)	1.79 (1.64–1.94)	1.81 (1.60–2.02)	2.14 (1.85–2.44)
No physical activity	3.19 (2.68–3.70)	3.19 (2.66–3.72)	3.20 (2.64–3.77)	4.12 (3.38–4.85)	4.20 (3.64–4.77)
Mediterranean diet followed	1	1	1	1	1
Mediterranean diet not followed	2.26 (1.95–2.58)	2.65 (2.27–3.04)	2.35 (2.00–2.71)	2.39 (2.03–2.76)	2.42 (2.15–2.70)
FLI low	1				
FLI moderate	5.47 (4.45–6.50)				
FLI high	10.13 (8.90–11.37)				
HSI normal		1			
HSI high		11.13 (9.93–12.34)			
ZJU normal			1		
ZJU high			9.88 (8.60–11.17)		
FLD normal				1	
FLD high				12.10 (10.80–13.51)	
LAP normal					1
LAP high					8.75 (7.56–9.95)

FLI Fatty Liver Index. HSI Hepatic Steatosis Index. ZJU Zhejian University. FLD fatty liver disease. LAP Lipid Accumulation Product.

**Table 4:** Areas under the curve, cutoff, sensitivity, and specificity of Youden index by sex.

	Women	Men
	AUC (95% CI)	AUC (95% CI)
FLI high	0.900 (0.884–0.916)	0.833 (0.817–0.848)
HSI high	0.878 (0.866–0.890	0.799 (0.785–0.814)
ZJU high	0.898 (0.888–0.909)	0.852 (0.838–0.865)
FLD high	0.935 (0.925–0.945)	0.917 (0.903–0.932)
LAP high	0.864 (0.848–0.881)	0.802 (0.786–0.818)
	cut-off-sens-specif-Youden	cut-off-sens-specif-Youden
FLI high	-2–83.5–83.4–0.669	-3–80.1–80.1–0.602
HSI high	-3–81.0–80.9–0.619	-5–77.0–74.1–0.511
ZJU high	-1–83.3–83.3–0.666	-3–81.0–77.5–0.585
FLD high	9–88.2–87.7–0.759	6–87.5–86.1–0.736
LAP high	1–80.4–80.3–0.607	-3–75.6–72.7–0.483

FLI Fatty Liver Index. HSI Hepatic Steatosis Index. ZJU Zhejian University. FLD fatty liver disease. LAP Lipid Accumulation Product.

## Data Availability

The study data are securely stored in a database that adheres to all security protocols at ADEMA-Escuela Universitaria. The designated Data Protection Officer is Ángel Arturo López González.
